# Role of viruses in asthma

**DOI:** 10.1007/s00281-020-00781-5

**Published:** 2020-01-27

**Authors:** Tuomas Jartti, Klaus Bønnelykke, Varpu Elenius, Wojciech Feleszko

**Affiliations:** 1 Department of Paediatrics, Turku University Hospital and University of Turku, Turku, Finland; 2 COPSAC, Copenhagen Prospective Studies on Asthma in Childhood, Herlev and Gentofte Hospital, University of Copenhagen, Copenhagen, Denmark; 3 Department of Pediatric Pneumonology and Allergy, The Medical University of Warsaw, Warsaw, Poland

**Keywords:** Asthma, Bronchiolitis, Child, Exacerbation, Genetics, Pathogenesis, Respiratory syncytial virus, Rhinovirus, Risk, Virus, Wheeze, Wheezing

## Abstract

Respiratory viral infections are the most important triggers of asthma exacerbations. Rhinovirus (RV), the common cold virus, is clearly the most prevalent pathogen constantly circulating in the community. This virus also stands out from other viral factors due to its large diversity (about 170 genotypes), very effective replication, a tendency to create Th2-biased inflammatory environment and association with specific risk genes in people predisposed to asthma development (*CDHR3*). Decreased interferon responses, disrupted airway epithelial barrier, environmental exposures (including biased airway microbiome), and nutritional deficiencies (low in vitamin D and fish oil) increase risk to RV and other virus infections. It is intensively debated whether viral illnesses actually cause asthma. Respiratory syncytial virus (RSV) is the leading causative agent of bronchiolitis, whereas RV starts to dominate after 1 year of age. Breathing difficulty induced by either of these viruses is associated with later asthma, but the risk is higher for those who suffer from severe RV-induced wheezing. The asthma development associated with these viruses has unique mechanisms, but in general, RV is a risk factor for later atopic asthma, whereas RSV is more likely associated with later non-atopic asthma. Treatments that inhibit inflammation (corticosteroids, omalizumab) effectively decrease RV-induced wheezing and asthma exacerbations. The anti-RSV monoclonal antibody, palivizumab, decreases the risk of severe RSV illness and subsequent recurrent wheeze. A better understanding of personal and environmental risk factors and inflammatory mechanisms leading to asthma is crucial in developing new strategies for the prevention and treatment of asthma.

## Introduction

Approximately 8–9% of children and adults suffer from asthma in Europe, and it is estimated that an equal number of individuals have asthma-like symptoms [1]. Fortunately, in the majority of patients, asthma is mild, but severe asthma occurs in 5–10% of them [2]. The early stages of asthma, bronchiolitis, or early wheezing affect 10–30% of children depending on definition and 15–25% of children experience recurrent wheezing before school age [3]. Thus, wheezing illnesses and asthma pose a significant health and socioeconomic burden in the world.

Although substantial progress has been made in understanding pathogenetic mechanisms of asthma, its risk-/protective factors, phenotypes, triggers, and differences between children and adults, we still do not truly understand nor can prevent it. Respiratory virus infections appear to be mutual and common triggers; the detection rate has reached 100% among young wheezing children decreasing to 80% in adults [4–6]. Especially, rhinovirus (RV), the common cold virus, is clearly the most common single trigger of exacerbations. It covers up to 76% of exacerbations of wheezing children and up to 83% of adults with asthma [4, 5]. Moreover, it is currently the most potent objective risk factor of school-age asthma among young wheezing children, odds rations (OR) reaching 45 depending on cofactors such as aeroallergen sensitization [7]. Most importantly, RV etiology in first-time wheezing children has served as an effective selection criterion for corticosteroid responders. In two trials, these children have responded to short-course of oral corticosteroid not only by effectively decreasing recurrent wheezing within the subsequent 12 months [3, 8] but also decreasing the incidence of asthma by 30% in a 4–7 years follow-up [9, 10]. These are the only trials that have shown that we can influence the natural course of asthma. Similar but not so marked treatment responses have also been shown in infants suffering from the respiratory syncytial virus (RSV)–induced bronchiolitis. In these patients, anti-RSV monoclonal antibody, palivizumab, has decreased the risk of severe RSV illness but also subsequent recurrent wheezing in long-term follow-up [11].

Whether respiratory viruses are causative agents of asthma or just secondary to a certain underlying condition, they are at least clinically relevant revealing factors of biased inflammatory mechanisms. By understanding the factors that increase susceptibility to virus infections and virus-induced inflammatory mechanisms in asthma, we are likely to better understand the pathogenesis of asthma and to identify novel targets for preventive strategies. The aim of this article is to review the role of virus infections in the pathogenesis of asthma inception and exacerbations, as well as to discuss interrelated protective and risk factors of asthma and treatment options.

## Virus etiology from bronchiolitis to asthma

Respiratory virus infections play a significant role in all wheezing (defined as a bilateral whistling sound during expiration accompanied by dyspnea) illnesses from bronchiolitis to asthma. Virus detection rates using PCR have reached 100% in bronchiolitis, 85–95% in children with recurrent wheezing or asthma exacerbation, and 80% in adults with asthma exacerbation [3]. The virus coinfection rate is typically 10–40% being more common in young children [12]. However, only a few studies support their association with a more severe clinical course [13]. It should be noted that virus detection rates using PCR may be over 35% level in asymptomatic subjects, especially in young children which sometimes question the significance of virus detection [3].

**Fig. 1:**
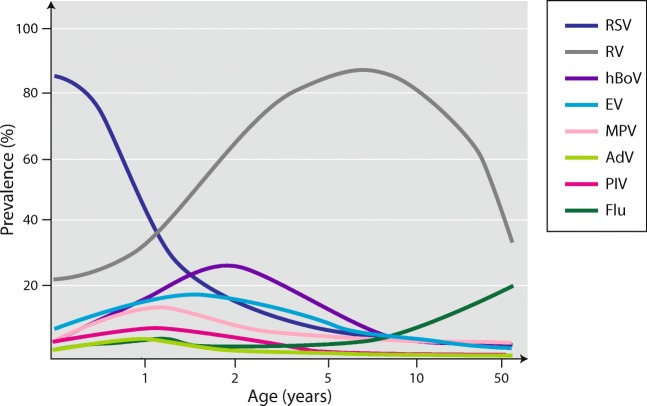
**Virus etiology from bronchiolitis and childhood wheezing to asthma**. RSV, respiratory syncytial virus; RV, rhinovirus; hBoV, human bocavirus; Flu, influenza virus; EV, enteroviruses; MPV, metapneumovirus; PIV, parainfluenza virus; AdV, adenovirus

According to the majority of guidelines, bronchiolitis is generally defined as virus infection of the lower respiratory tract (bronchioles and their surrounding tissues) in children less than 2 years of age [14]. RSV is the most important causative agent during infancy, and its detection rates range 50–80% in hospitalized cases (Fig. 1) [12, 13]. RV is the second most common etiologic agent during infancy (and the most common outside RSV epidemics), but it starts to dominate virus detection approximately after 12 months (Fig. 1) [12–14]. The next most common etiologic viruses are human bocavirus and human metapneumovirus (detection rates may reach 10–25%) followed by the parainfluenza virus, adenovirus, coronavirus, and influenza virus each of which typically make less than 10% (Fig. 1). In children with recurrent wheezing and asthma exacerbation, as well as adults with asthma exacerbation, RV clearly dominates as a trigger [4, 12]. Its detection rate has reached 76–83% in adults.

### Rhinovirus

Rhinovirus is a non-enveloped positive-strand RNA virus in the family Picornaviridae, genus Enterovirus, and is classified into three species (RV-A, B, and C) [3]. There are over 170 distinct RV genotypes, including 80 RV-A, 32 RV-B, and 65 RV-C genotypes. The RV-C group was not found until 2006 (approximately 50 years after the first discovery of other RVs) since it does not grow in conventional cell cultures. Currently, PCR is the method of choice in identifying RVs from nasal secretions. We are moving towards RV species specific diagnosis since RV-A and RV-C appear to cause the more severe course of illness than RV-B [15]. Nevertheless, the common cold is the main type of illness. Up to 35% of asymptomatic subjects may be positive for RV [3]. RV does not cause chronic infection or “colonization” in healthy individuals [3]. RV circulates year-round with multiple coexisting genotypes that potentiate its asthmagenic activity [3]. Peak prevalence in temperate climates occurs in the early autumn and late spring. In exacerbations of asthma, RV-A and RV-C are clearly more common etiologic RV species than RV-B—similar to wheezing illnesses in early childhood [16, 17].

RV-C has also been the most common RV species among hospitalized and intensive care unit asthma patients [18]. Patients with CDHR3 (cadherin-related family member 3) asthma risk allele and atopic individuals have been more susceptible to RV-C infections (see below) [19, 20]. At the cellular level, RV-C has caused faster replication rate and induction of more robust cellular responses than RV-B, as demonstrated in cultures of differentiated airway epithelial cells [3]. Overall, the clinical impact of RV-A is close to RV-C among asthmatic subjects, whereas the impact of RV-B is much weaker. Cohort studies have, however, shown that RV-B infections may slightly increase the risk of exacerbation in children whose asthma is of greater severity [3, 21].

### Respiratory syncytial virus and other viruses

As mentioned above, other species than RV do not appear to be of major significance as triggers of asthma. Typically, the first infection with RSV or with its close relative, metapneumovirus, may be severe and cause bronchiolitis [14]. However, reinfections, which typically occur annually, are mild. Human bocavirus seconds to RV in the age group of 2–5-year-olds cause severe lower airway illnesses and by the age of 5 years, practically all children have acquired protective antibodies [6]. Influenza virus infection may contribute to asthma attacks in adults, but due to effective vaccination programs, they have become rare [4, 22]. Coronaviruses (excluding SARS and MERS), parainfluenza viruses, adenoviruses, and respiratory polyomaviruses (KI and WU) typically cause just upper airway infections.

## Genetics and epigenetics of asthma and virus susceptibility

Asthma is a highly heritable disease with heritability estimates from twin studies of more than 50%, and even higher for childhood asthma [23]. Genetics is, therefore, one important tool for understanding the disease mechanisms of asthma. Our knowledge of the genetic background of asthma has increased rapidly during recent years through methodological advances allowing so-called genome-wide association studies (GWAS) where millions of genetic variants covering the entire genome can be tested without a prior hypothesis about underlying mechanisms. Large GWAS have now identified more than 100 genes/loci associated with asthma and related traits, with the strongest signal seen for the childhood-onset disease [24]. Of these, the majority of susceptibility genes are related to the immune system.

The first asthma locus discovered in GWAS, approximately 10 years ago, was the chromosome 17q21 locus [25]. It is still the strongest known asthma locus, and it is particularly associated with childhood-onset asthma and asthma with severe exacerbations [26]. Interestingly, the 17q21 asthma locus is strongly associated with increased risk of early wheezing episodes triggered by viruses, including rhinoviruses, and children with early viral wheezing have a much higher risk of later asthma if they carry 17q21 risk variants [27]. Also, 17q21 risk variants seem to interact with several environmental factors so that children with risk variants are more susceptible to having older siblings in terms of increased risk of early wheeze, but also more protected against asthma when exposed to a farming environment or pets in the household in early life [28]. The biological mechanisms associated with the 17q21 locus are still incompletely understood and might involve several genes in the region, including *ORMDL3*, *GSDMB*, *GSDMA*, *PGAP3*, *ERBB2*, and *IKZF3* and several cell types, including immune and airway epithelial cells [26]. Most follow-up studies have focused on *ORMDL3* as the causal gene with proposed mechanisms related to sphingolipid synthesis [29], regulation of eosinophils [30], and regulation of the ICAM1 receptor, which might explain the susceptibility for rhinovirus infections [31]. Further understanding of the biological pathways related to this locus might provide important novel clues to the mechanisms of viral infections and asthma.

Another asthma risk gene, *CDHR3*, was discovered in a GWAS of childhood asthma with recurrent acute hospitalizations before 6 years of age [32]. Experimental studies have later demonstrated that CDHR3 functions as a rhinovirus-C receptor and is highly expressed in differentiated bronchial epithelial cells [33]. The association between *CDHR3* risk variants and rhinovirus-C respiratory illness was subsequently confirmed clinically in birth cohorts [19]. These findings suggest that the mechanism associated with *CDHR3* variants is at least partly explained by an increased risk of rhinovirus-C respiratory illnesses and that targeting CDHR3 might be a strategy for preventing rhinovirus-C triggered asthma exacerbations.

Genetic discoveries in GWAS require a very large number of participants, and large well-powered GWAS of respiratory viral infections per se have not yet been performed. One smaller GWAS of bronchiolitis was performed without genome-wide significant findings [34]. Candidate gene studies, most focusing on RSV bronchiolitis, have suggested several susceptibility genes related to immune regulation and surfactant proteins [35]. Several of these genes have also been associated with asthma, indicating that the association between RSV bronchiolitis and later asthma development might partly be explained by shared genetics.

Epigenetics, defined as heritable changes in gene expression without changes in the underlying DNA sequence, is one mechanism by which environmental factors can affect gene regulation and may explain long-term programming of disease from early life exposures and changes in disease status time. The most commonly studied epigenetic mechanism is DNA methylation. A large genome-wide study on methylation indicated the role of methylation in eosinophils in the pathogenesis of asthma [36]. Since methylation is tissue-specific, it might be crucial to study the relevant “target-organ,” and two genome-wide studies of methylation in nasal epithelium have found several differentially methylated sites associated with allergic sensitization and asthma [37, 38].

In order to understand the genomics of asthma and virus infections, we need more studies that combine information on gene variants, gene expression, and epigenetics in relevant cells and also assessed during acute symptoms with information on specific infectious triggers. Such studies are challenging, but also have the potential to reveal unknown disease mechanisms and functional subtypes of the disease, which is essential in order to personalize and improve treatment and prevention of disease.

## Risk factors of asthma

**Fig. 2:**
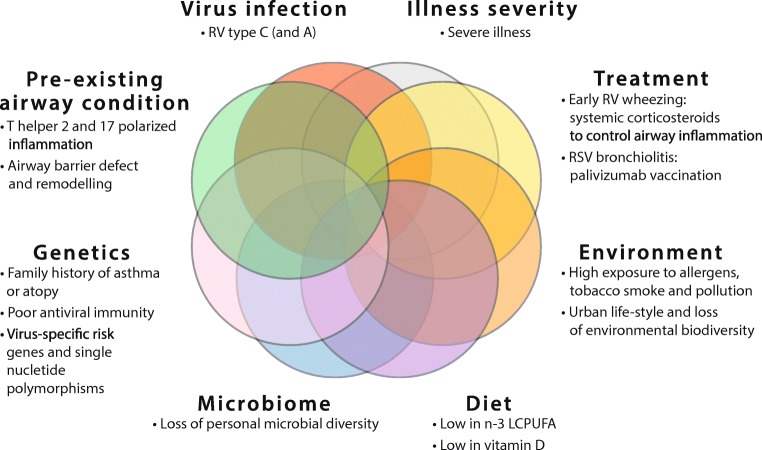
**Major factors influencing asthma risk in young children suffering from bronchiolitis**. RV, rhinovirus; virus; n-3 LCPUFA, n-3 (omega-3) long-chain polyunsaturated fatty acids

Severe acute bronchiolitis or early wheezing is associated with an increased risk of subsequent asthma [3]. This disease may persist until early adulthood, and the strongest association with worse long-term prognosis was observed in children infected with RV-C and RV-A virus (Fig. 2) [39]. These observations come from cohort (population) studies, where the most striking relationship was recorded in children at high risk, i.e., in patients hospitalized due to severe disease, in children from atopic families, and children with atopy (Fig. 2) [3]. Coexpression of aeroallergen sensitization or 17q21 asthma risk alleles increased the odds ratios to the level of 20–45 (Fig. 2) [7, 27]. Collectively, these findings suggest that RV is most likely a revealing factor for those with early airway inflammation (i.e., broken epithelial barrier, T helper_2_ cell polarized immune events), genetic variation children (i.e., may markedly increase the risk of asthma), and/or low interferon responses (i.e., impaired viral defense) and therefore serves as a clinically useful risk marker [7, 19, 27, 40, 41].

Before the discovery of the link between RV-induced bronchiolitis and later asthma, it was long thought that RSV is the key “asthma-inducing” pathogen (Fig. 2). Several studies have reported an association between RSV bronchiolitis and school-age asthma but not with atopy, excluding one relatively small cohort study [3]. However, odds ratios have been at a relatively low level (OR 2.5–4.5), and these studies were limited in not investigating RV etiology. Similar risk numbers have also been found in pre-term infants, which are also more susceptible to severe RSV infections [14]. Other risk factors include young age, parental smoking, and common asthma risk genes [3, 14]. Unlike with RV, RSV causes more direct damage to the airway epithelium, but still, the common perception is that RSV infection is not causal to asthma or atopy development. Children with RSV infection are likely to share common genetic vulnerability and/or environmental exposures that predispose them to both diseases (Fig. 2) [3, 14].

We have recently reviewed the main non-viral risk and protective factors of asthma and summarized in Fig. 2 [3, 14]. Rapid urbanization, pollution, and climate change, all leading to the loss of biodiversity, promote chronic non-communicable illnesses such as asthma and allergies [42]. Exposure to pollutants such as NO_2_ and high exposure to allergens in children with allergic asthma increase the severity of virus-induced exacerbations of asthma. Maternal stress and depression have been associated with acute wheezing illnesses. Of dietary ingredients, low maternal omega-3 long-chain polyunsaturated fatty acid (n-3 LCPUFA) has increased the risk of persistent wheeze/asthma in offspring [43]. Low vitamin D levels increase susceptibility to virus-induced wheezing and severity of asthma. However, recommended supplementation has mainly abolished its effect [44]. Common protective factors of allergy and asthma include a healthy lifestyle (healthy nutrition, exercise, outdoor activities), diverse personal microbiome, non-polluted air, and environmental biodiversity, as well as contact with animal and farm dust.

## Viruses and microbiome

As described above, there is evidence of a viral trigger in most acute asthma episodes. However, also, bacteria might play an important role in the pathology of acute asthma symptoms. As an example, young children with acute wheezy episodes had increased detection rates of the bacterial pathogens *Moraxella catarrhalis*, *Streptococcus pneumoniae*, and *Haemophilus influenzae* in hypopharyngeal aspirates [45]. Indirect evidence of a potential causal role of bacteria for such acute symptoms comes from findings that treatment with the antibiotic azithromycin reduced the symptom burden of such episodes [46, 47], although this can also be due to anti-viral or anti-inflammatory effects of macrolides.

Similar to viruses, airway bacteria have also been suggested as early life risk factors for later development of asthma, as indicated by an association between the detection of pathogenic bacteria in the airways of asymptomatic infants and later development of asthma [48]. Also, the gut microbiome, a putative risk factor for asthma, might influence the susceptibility to viral infections in the airway as indicated by a mouse model where supplementing with *Lactobacillus johnsonii* protected against RSV infection [49].

In addition, there is substantial evidence of the interaction between airway bacteria and viruses. In a study of acute respiratory symptoms in healthy and asthmatic children, rhinovirus was associated with increased detection of bacterial pathogens, and *Moraxella catarrhalis* and *Streptococcus pneumoniae* seemed to contribute to the severity of respiratory tract illnesses and asthma exacerbations [50]. At the epidemiological level, the seasonal peaks of several viral infections are associated with increased hospital admission for invasive *Streptococcus pneumoniae* disease [51]. There are several mechanisms by which viral infections can increase the risk of bacterial infections, including immune suppression, epithelial damage, and changes in the local lung environment altering the growth conditions for pathogenic bacteria. But also the other way around, bacterial infection and/or colonization of the airways might increase the risk of later viral infection. Vaccination against *Streptococcus pneumoniae* in a randomized trial resulted in subsequent reduced risk of virus-associated pneumonia [52]. There is also evidence of interaction between viruses and the microbiome in studies using a broader sequencing-based characterization, whereby both culturable and non-culturable bacteria can be detected. One study found an association between a nasopharyngeal microbiome characterized by *Haemophilus influenzae* and *Streptococcus* and RSV hospitalization [53], and another study found that a nasopharyngeal microbiome dominated by *Streptococcus* in asymptomatic infants was associated with later wheezing [54]. These findings emphasize the importance of assessing both bacteria and viruses in studies of asthma etiology in order to address their individual roles and the impact of their interaction.

## Pathogenesis of asthma

Asthma is a syndrome characterized by intermittent attacks of breathlessness, wheezing, and cough accompanied by variable airflow obstruction. Asthma is now considered an umbrella diagnosis for several diseases with distinct mechanistic pathways (endotypes) with variable clinical phenotypes (childhood atopic, non-atopic, middle-aged obese, and elderly late-onset). An important molecular mechanism of asthma is the chronic inflammation of conducting airways, even during asymptomatic periods. This inflammation is different in various asthma endotypes and may broadly be divided into Th2 high (atopic, eosinophilic) and Th2 low (non-atopic, non-eosinophilic) endotypes [55].

**Fig. 3:**
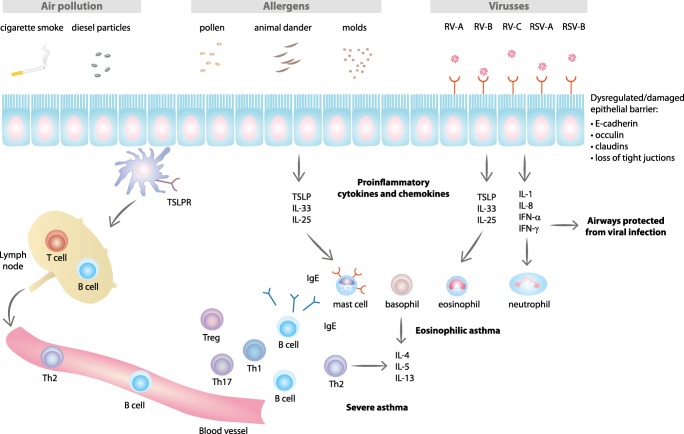
**Airway epithelial pathways impacted by environmental exposures and type 2 immune responses in asthma pathogenesis**. Exposure to air pollutants (cigarette smoke, diesel particle, etc.) cause oxidative stress. Mold and other allergens stimulate epithelial cells and induce activation of proinflammatory cytokines and chemokines. Respiratory viruses (RV and RSV) interact with specific receptors on epithelial cells. Damaged or dysregulated epithelial barrier in asthmatic patients also affects type 2 inflammation. Release of cytokines and chemokines promotes activation and mobilization of type 2 immune responses. CDHR3, cadherin-related family member 3; CX3CR1, CX3C chemokine receptor 1; ICAM-1, intercellular adhesion molecule 1; IFN, interferon; IgE, immunoglobulin E; IL, interleukin; LDLR, low-density lipoprotein receptor; RV-A, RV-B, RV-C, rhinovirus-A, rhinovirus-B, rhinovirus-C; RSV-A, RSV-B, respiratory syncytial virus-A, virus-B

Type 1 and type 2 immune responses are regulated by T helper 1 (Th1) and 2 (Th2) cells, respectively. Th1 cells secrete IL-2 and IFN-γ and stimulate type 1 immunity characterized by phagocytic and anti-viral activity [56]. Th2 cells mainly secrete inflammatory cytokines such as IL-4, IL-5, and IL-13 that stimulate Th2 type immunity characterized by eosinophilia and high antibody titers (Fig. 3) [57]. Th2 type immune response is induced by parasites, but it is also associated with the atopic disease; allergy, allergic rhinitis, and asthma [58]. Th2 type responses are mediated by eosinophils, basophils, mast, Th2, and IgE-producing B cells (Fig. 3). Increased production of type 2 cytokines leads to allergen-triggered IgE hypersensitivity and activation of mast cells, basophils, eosinophils, airways epithelial cells, and remodeling of airways (Fig. 3).

**Fig. 4:**
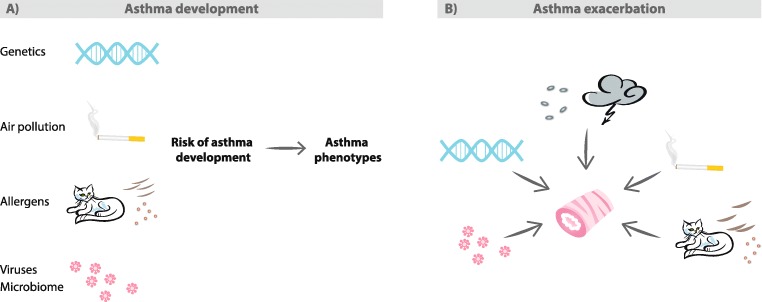
**Summary of environmental factors affecting developmentaand exacerbationsbof asthma**. **A**) Interplay of environmental (air pollution, allergens, viruses) and host (genetic, microbiome) factors shape the risk of asthma development and predispose to different asthma phenotypes. **B**) Environmental exposures to allergens (animal, pollen, mold), viruses, cigarette smoke, and air pollution are known triggers for asthma exacerbations. Thunderstorms are associated with asthma exacerbations. Thunderstorms produce ozone and release allergen-bearing small particles that irritate airways. Avoidance of environmental exposures can improve asthma control and reduce exacerbations

Childhood asthma is often associated with other allergic diseases, such as atopic eczema and allergic rhinitis. Th2 type inflammation in the airways often starts in childhood, when environmental stimuli such as viral respiratory tract infection, exposure to parental smoking, and NO_2_ and other airborne pollutants or allergens activate airway epithelial cells to produce type 2 inflammatory cytokines including IL-25, IL-33, or TSLP (thymic stromal lymphopoietin) (Fig. 4) [56]. This initiates a cascade that leads to the development of childhood asthma (Figs. 3 and 4). The reason why this Th2 type response persists in some patients is not well known.

Having allergic sensitization and eczema at the time of wheezing with rhinovirus are all risk factors of having atopic asthma at the age of 7 years [59]. On the contrary, having RSV as the cause of the first wheeze before the age of 1 year or exposure to parental smoking are both associated with non-atopic asthma at age 7 years [59].

A recent study showed that the risk of developing asthma was highest in infants having IgE sensitization and wheeze due to rhinovirus-C infection [39]. These results suggest that mechanisms of virus-induced illnesses differ. The mechanisms underlying the observed associations between rhinoviruses, allergic sensitization, and the development of asthma are not fully understood. It is possible that there is a causal relationship where acute rhinovirus infection induces various cellular factors regulating host response, airway inflammation, repair, and remodeling, as well as increase proinflammatory cytokine and chemokine production (Fig. 4) [60, 61]. Also, rhinovirus infection and allergen exposure increase epithelial cells to produce IL-25 and IL-33, thus promoting Th2 type inflammation (Fig. 3) [62–64]. Alternatively, virus infection can simply be an early marker of impaired anti-viral response; diminished type I and III interferon production and/or abnormal host response; induced expression of IgE receptors [65] or genetic susceptibility to rhinovirus infection due to enhanced expression of rhinovirus-C receptor CDHR3 in the airway epithelium [32]. Most likely, all of these mechanisms play a role in the development of either atopic or non-atopic asthma.

## Treatment of viral wheeze to prevent asthma

**Fig. 5:**
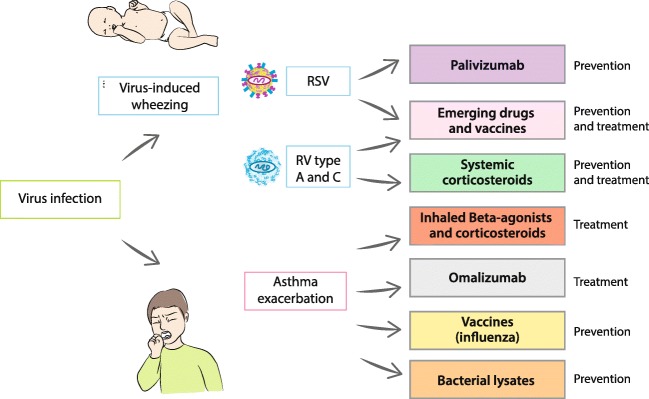
**Current strategies for asthma prevention and treatment**. Details in text. RV, rhinovirus; RSV, respiratory syncytial virus

Several therapeutic strategies have been shown to alter the natural history of virus-induced asthma exacerbations (Fig. 5). In general, such treatment would need to be applied as early as possible during infection to increase the chances of success, safety, and be easy to administer.

### Prevention

Currently, there exist no safe and effective human vaccines for RSV nor RV. The major obstacle is an antigenic diversity of the more than 100 serotypes of rhinovirus, meaning that creating a successful vaccine is an extremely challenging task [66]. In the development of the RV vaccine, promising results have been seen with a cross-reactive recombinant capsid protein in a mouse model [67]. Recently, attention has been caught to live attenuated vaccines and subunit vaccines against RSV combined with Th1-enhancing adjuvant, although neither of them seems likely to be introduced to routine clinical practice soon [68].

### Prevention and treatment of RSV

#### Palivizumab

A humanized monoclonal antibody against the RSV fusion (F) protein, currently used for immunoprophylaxis was proved to decrease the risk of hospitalization due to severe RSV illness among pre-term infants (72% reduction), those with chronic lung disease (65% reduction), and hemodynamically significant congenital heart disease (53% reduction) [68]. The application of palivizumab resulted in a remarkably reduced risk of recurrent wheezing episodes following hospitalization due to RSV, but not asthma [11]. Interestingly, a new, second-generation high-affinity derivative of palivizumab (motavizumab) did not prevent long-term recurrent wheezing despite reducing the rate of severe acute RSV disease [69].

#### Ribavirin

There is no convincing data supporting ribavirin treatment for severe RSV infection, while there are concerns about its toxicity. Therefore, ribavirin is neither recommended in the USA nor any European guidelines [70].

#### New approaches

At this time, there are 17 new RSV vaccines and biologicals in a pipeline of clinical trials while another 28 are in pre-clinical development [68]. Numerous new molecules have already been characterized as capable of inhibiting RSV dissemination within the airways and are investigated as potential candidates for pre-clinical and clinical development [66].

### Prevention and treatment of rhinovirus

#### Drugs (pleconaril, amantadine, rimantadine)

Although it is hypothetically possible nowadays to interfere with every step of the infectious cycle of respiratory tract viruses (from viral attachment, viral entry and uncoating, translation, replication, and onward to virus release), only a few approaches have met with success with RV thus far. There is only a limited number of agents that interact with the RV attachment to the cell or uncoating of the viral RNA that have been tested in clinical trials (pleconaril, amantadine, rimantadine) [71]. Regrettably, their clinical applicability is continuously questioned due to adverse events (pleconaril, vapendavir) or drug resistance (amantadine, rimantadine) [66].

#### Prednisolone

According to current evidence, early systemic anti-inflammatory management may drastically affect the natural course of asthma development, probably via targeting pre-existing Th2-skewed immunity and/or virus-induced airway inflammatory response.

Two separate randomized trials exist, in which oral corticosteroid, prednisolone, has been applied to wheezing children with RV etiology. Intriguingly, prednisolone application was shown to decrease the time to the physician-confirmed relapse within the following year (by 20–30%) and time to the initiation of asthma controller medication within the following 5 years (by 30–40%) in these children [8, 9, 72, 73]. Noteworthy, high RV genome load in the placebo-treated wheezing children was associated not only with the development of a new wheezing episode within 100 days in every case but also with the initiation of asthma medication within the next 14 months in every case [72, 73]. These results indicate that systemic anti-inflammatory treatment of the first RV-induced severe wheezing episode may markedly decrease the subsequent risk for asthma.

### Long-term sequela

Whether RV bronchiolitis is the cause of severe lung injury, resulting in subsequent wheezing episodes and development of asthma or if there is an inborn susceptibility to both acute bronchiolitis and subsequent asthma remains still a matter of debate. Nevertheless, the major viral causes of acute bronchiolitis/first wheeze are RSV and RV, which seem to have a different course in post-bronchiolitis asthma sequela, apart from underlying lung morbidity (for review, see Jartti et al. [14]).

Subsequently, two prospective studies with RSV immunoprophylaxis have been performed to address the potential causality between RSV infection and subsequent asthma. In these recent randomized controlled trials, pre-term infants who received palivizumab demonstrated a decreased number of recurrent wheezing episodes, but the incidence of physician-diagnosed asthma at age 6 remained intact [74, 75]. These effects, however, were less noticeable in infants with atopic family history, indicating that RSV infection is not causal to asthma or atopy development.

On the contrary, atopy is associated with childhood asthma inception after RV-induced bronchiolitis. A study in high-risk birth cohort (parental atopy or asthma) from WI, USA, has demonstrated that in young children who experienced RV-induced bronchiolitis, there is a high risk of school-age asthma (OR 9.8 vs. 2.6; RV vs. RSV), and the risk becomes even higher in children sensitized at an age younger than 2 [7, 76]. Another study from Turku, Finland, shows strikingly similar results. In infants at the age of less than 2 years, who developed RV-induced bronchiolitis, the odds ratio for atopic asthma at school age was 5.0, which increase up to 12 when combined with early sensitization [59].

RSV-induced bronchiolitis was neither associated with atopic nor non-atopic asthma [59]. Altogether, the above-presented data suggest that airways in “high-risk” individuals display an increased susceptibility for asthma inception after RV-induced bronchiolitis [77]. Protection of these high-risk children against the effects of severe respiratory infections during infancy may represent an effective strategy for primary asthma prevention.

## Mechanisms of asthma exacerbation

Asthma exacerbation is defined as an acute or subacute worsening of asthma symptoms and lung function as compared to the patients’ usual health status. Exacerbations usually occur in response to a variety of external agents, including respiratory viruses, bacteria, allergens, air pollutants, smoke, and cold or dry air (Fig. 4). However, it is estimated that up to 85–95% of asthma exacerbations in children and 75–80% in adults are linked to viral infections. However, most viral infections are not associated with acute exacerbations, and cofactors, including bacterial and allergic inflammation, have been described to increase the severity of exacerbation (Fig. 4). The most common viral triggers for asthma exacerbation are rhinoviruses, particularly subtypes A and C [15]. Hospital admissions for asthma exacerbations correlate with a seasonal increase of RV infections in autumn from September to December and again in spring [15, 78].

Other respiratory viruses may also cause exacerbations. Respiratory syncytial virus (RSV), which frequently causes wheeze in infants and young children, can also trigger acute asthma exacerbation in adults [79]. Human metapneumovirus, influenza, parainfluenza, adenovirus, coronavirus, and bocavirus have all been detected in asthma exacerbations but in lower frequencies [3].

Several mechanisms why asthmatics are predisposed to viral infections have been proposed. One of the proposed reasons is damaged epithelium, which may increase susceptibility to infection and ultimately lead to airway obstruction (Fig. 3) [80]. Cellular mechanisms involved in viral response include disruption of the epithelial barrier and tight junctions, impaired apoptosis, increased cell lyses, deficient Th1 response and reduced IFN-γ production, and enhanced expression of viral receptors (Fig. 3) [80]. Viral infection also induces proinflammatory cytokines, including interleukins as well as mediators or remodeling (VEGF, FGF) (Fig. 3) [71].

Viruses attach to their unique cellular receptors: intercellular adhesion protein 1 (ICAM-1) is used by the majority of RV-A and all RV-B types, low-density lipoprotein receptor family members (LDLR) are used by RV-A, cadherin-related family member 3 (CDHR3) is used by RV-C, and CX3CR1 is used by RSV (Fig. 4) [33, 81, 82]. Interestingly, the corresponding gene for CDHR3 has been linked to childhood asthma with severe asthma exacerbations [32].

Airway epithelial cells form a barrier to the outside world and are the front line of mucosal immunity (Fig. 4). Cytokines and inflammatory mediators associated with allergic inflammation induce epithelial barrier disruption. Additional factors that influence the severity of the viral infection and the risk of asthma exacerbation are allergic sensitization and the airway microbiome. For example, RV infection and allergic sensitization synergistically increase the risk of exacerbation [3]. Atopic asthma with allergic sensitization can be associated with reduced virus-induced IFN responses, increased viral shedding, and decreased viral clearance [3].

Moreover, bronchial epithelial cells of atopic asthmatics have shown to have increased RV replication, deficient IFN-γ release, and enhanced cell lysis [83]. Studies with omalizumab, anti-IgE, have shown that neutralizing IgE-mediated inflammation can enhance IFN responses and reduce virus-induced asthma exacerbations in children [84]. This finding suggests that neutralizing IgE indirectly improve anti-viral responses.

Several studies have shown that viral infections precede bacterial infections in airways. This phenomenon may occur due to several reasons; viruses may induce expression of airways receptors used by bacteria, viruses may disrupt epithelial barrier, and they increase the release of inflammatory cytokines and mediators causing increased inflammation and risk of asthma exacerbations (Fig. 4) [85]. In addition, patients with asthma are frequently colonized with bacteria in lower and upper airways [48], and acute wheezing in infants has been associated with both viral infections and airway microbiota dominated by bacterial pathogens [50]. Bacterial infections may impair mucociliary clearance and increase mucus production. However, evidence linking bacterial infections to acute asthma exacerbation is limited.

## Treatment of asthma in regard to viral infections

Exacerbations of asthma are characterized by a progressive increase in symptoms of shortness of breath, cough, wheezing, and progressive decrease in lung function. Viral respiratory infections remain a leading cause of asthma exacerbations, both in children and adults. The presence of any pathogen is usually associated with a higher risk of treatment failure [86]. Typically management of all asthma exacerbations includes a symptomatic treatment increasing doses of beta 2-agonists, enhancing the use of inhaled or oral glucocorticosteroids [87].

### Glucocorticosteroids

Glucocorticosteroids are by far the most widely used drugs in children with asthma and have potent anti-inflammatory activity. In recent years, an increasing body of pre-clinical evidence supports their use in combination with long-acting beta-agonists, such as salmeterol and formoterol, and highlights the superiority of combination therapy in asthma exacerbations over either drug alone. Combination of either salmeterol and fluticasone or budesonide and formoterol treatment in vitro has been shown to synergistically suppress induction of several chemokines (CXCL8, CCL5, and CXCL10) and remodeling-associated growth factors (including FGF and VEGF) upon RV infection (for review see Jackson and Johnston [71]). The effective suppression of growth factors highlighted above certainly represents a plausible mechanism through which these drugs might inhibit virus-driven inflammation and remodeling.

### Omalizumab

Surprisingly, systemic anti-IgE treatment was also shown to markedly reduce infection-induced severe asthma exacerbations. A year-round treatment with omalizumab has been shown to abolish the seasonal peaks in asthma exacerbations, most of which are associated with RV infection [84]. Parallel to the IgE neutralization by the drug, clinical benefit was associated with enhanced IFN-γ responses, suggesting that omalizumab may improve the anti-viral responses [88].

### Influenza vaccine

Influenza contributes to some acute asthma exacerbations. Children with asthma should remain a priority group for influenza immunization because of the newly established association between influenza and ED management failure combined with well-recognized influenza-related complications [88]. This recommendation has been ultimately confirmed by a recent systematic review and meta-analysis, showing that influenza vaccination reduced the risk of asthma exacerbations [22].

### Immunomodulators and bacterial lysates

Among several non-specific anti-viral approaches to reduce asthma include strategies aiming at enhancing the patient’s resistance to multiple respiratory viruses through the administration of immunostimulatory preparations [66, 89]. Pidotimod is a synthetic thymic dipeptide that appears to share several mechanistic similarities with bacterial immunomodulators, and it is thought to stimulate toll-like receptor 2 (TLR2) and TLR4, which are expressed on DCs, this displaying anti-infective effects. To date, there is only one prospective multicenter trial, showing that pidotimod reduced the number of respiratory infections in a mixed group of children over half of whom had atopic conditions, including asthma [90].

Bacterial lysates have recently been proved to reduce the number of the recurrent wheezing episodes and asthma episodes, in patients treated with BL compared with placebo (5 trials) [91]. However, higher-quality trials are required before firm conclusions can be drawn regarding the prophylactic efficacy of bacterial lysates in asthma. A new large scale trial, currently carried out in the USA (ORal Bacterial EXtracts for the prevention of wheezing lower respiratory tract illness, ORBEX, NCT02148796; *N* = 1000) will address these concerns, but its results are expected in 2022.

### Anti-virals

There are several novel approaches, being tested in laboratories and clinical research for their ability to target RV-induced infection. These include soluble ICAM-1 receptor interfering with RV attachment (tremacamra), and two anti-viral drugs (pleconaril and ruprintrivir) as well as the application of inhaled IFN-β after the onset of a respiratory tract viral infection in asthmatic subjects. All of them, however, show only marginal benefit in symptoms, viral replication, and development of clinical symptoms of colds, while exhibiting substantial side effects [71].

### Conclusions

- There is an abundance of data showing that RV-C and RV-A contribute to asthma development and/or is a marker of asthma susceptibility.
- Using viral markers in relation to treatment might be a good strategy to prevent asthma.
- Oral application of corticosteroids may change the natural history of asthma.
- Treatment of virus-induced wheezing/asthma exacerbations with high-dose corticosteroids prevents destructive cytokine release in the airways.

Here we suggest that prevention and treatment of recurrent wheezing may in the foreseeable future be based on virological tests at the first episode of wheezing. Thereby, existing treatment methods (beta2-agonists and corticosteroids) may be more effective when given to a distinct (RV-affected) high-risk group of patients making treatment more personalized.
